# Isolation of new indole alkaloid triglucoside from the aqueous extract of *Uncaria rhynchophylla*

**DOI:** 10.1007/s11418-024-01836-9

**Published:** 2024-08-22

**Authors:** Yuta Koseki, Hiroaki Nishimura, Ryuji Asano, Katsuyuki Aoki, Li Shiyu, Ryosuke Sugiyama, Mami Yamazaki

**Affiliations:** 1 Tsumura Botanical Raw Materials Research Laboratories, Tsumura and Co., 3586, Yoshiwara, Ami‑machi, Inashiki‑gun, Ibaraki, 300‑1192 Japan; 2 Shenzhen Tsumura Medicine Co., No.99, Fuyon Road, Fuyong Street, Baoan District, Shenzhen, 518103 Guandong China; 3 Graduate School of Pharmaceutical Sciences, Chiba University, 1-8-1 Inohana, Chuo-Ku, Chiba, 260-8675 Japan; 4 Plant Molecular Science Center, Chiba University, 1-8-1 Inohana, Chuo-Ku, Chiba, 260-8675 Japan

**Keywords:** *Uncaria rhynchophylla*, Indole alkaloid triglucoside, Micro-drop, Single-crystal X-ray diffraction

## Abstract

*Uncaria rhynchophylla* (Miq.) Miq. (Rubiaceae) is widely used as a botanical raw material for traditional Japanese and Chinese medicines. However, not all of its potentially bioactive constituents have been isolated and characterized. Herein, one new indole alkaloid triglucoside (**1**), nine known alkaloids (**2**–**10**) and thirteen known non-alkaloids (**11**–**23**) were isolated from the aqueous extract of *Uncaria rhynchophylla* hook and structurally characterized ^1^H and ^13^C NMR and high-resolution electrospray ionization mass spectrometry. The absolute configurations of isolated compounds (**1**, **2** and **3**) were determined by the X-ray diffraction analysis of their single crystals obtained using a micro-drop crystallization technique. This technique allows single crystals to be obtained from samples as small as 50 µg, thus providing detailed structural information even on minor constituents and enabling the accurate quality monitoring of botanical raw materials more accurately.

**Graphical abstract:**

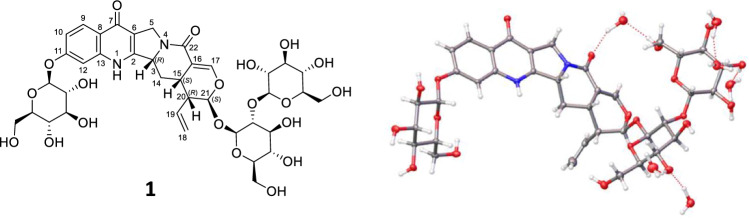

**Supplementary Information:**

The online version contains supplementary material available at 10.1007/s11418-024-01836-9.

## Introduction

**Fig. 1:**
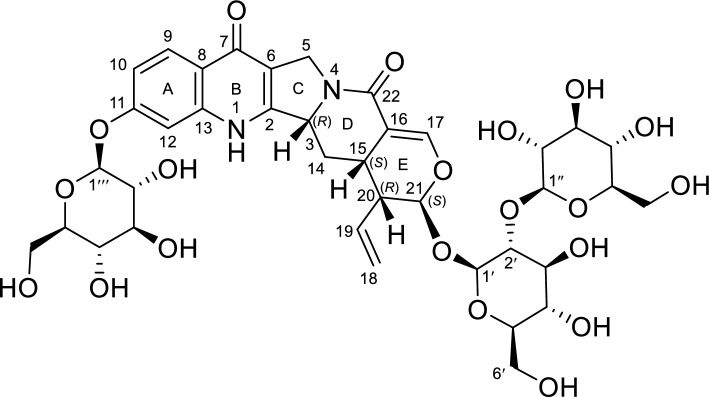
The structure of compound **1**

**Fig. 2:**
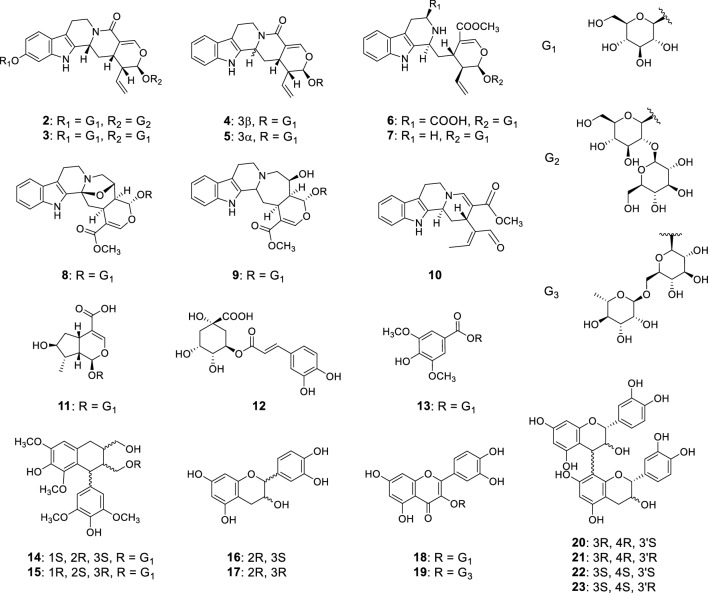
The structure of known compounds **2**–**23**

*Uncaria rhynchophylla* (Miq.) Miq. (Rubiaceae), an evergreen liana growing in warm climates and distributed in the southern part of the Boso peninsula in Japan and in central and southern China, is widely used as a botanical raw material for traditional Japanese and Chinese medicines. For example, a Kampo formula consisting of *U. rhynchophylla* hooks (Yokukansan) is used to treat neurosis, anxiety, night-time crying, and the behavioral and psychological symptoms of dementia [1]. Although the compounds contained in these hooks (alkaloids, triterpenes, phenolic acids, and flavonoids) have been extensively profiled for many decades, novel constituents continue to be discovered [2–6]. Given that the constant quality of traditional Japanese medicines can only be achieved when the quality of the corresponding botanical raw materials is secured, the identification of previously unreported *U. rhynchophylla* constituents is a matter of high practical significance. Herein, we profiled the aqueous extract of *U. rhynchophylla* hooks and and isolated one new alkaloid (**1**), nine known alkaloids (**2**–**10**), and thirteen known nonalkaloids (**11**–**23**) (Figs. 1 and 2). The structures of known compounds were determined by spectroscopic analysis (high-resolution electrospray mass spectrometry (HR-ESI–MS) and NMR spectroscopy). The absolute configuration of **1** was determined as rhynchophylloside L 11-*O-β*-D-glucopyranoside by single-crystal X-ray diffractometry (SC-XRD) using a micro-drop crystallization technique, the validity of which was confirmed by application to known compounds, namely rhynchophylloside G (**2**) [5] and vincosamide 11-*O*-*β*-D-glucopyranoside (**3**) [7].

## Results and discussion

*U. rhynchophylla* hooks (14.25 kg) were percolated with 60% (v/v) aqueous MeOH, and the aqueous phase obtained after the evaporation of MeOH was extracted with CHCl_3_ to remove hydrophobic constituents and fractionated by column chromatography (DIAION HP-20, silica gel, reversed-phase C18, Sephadex LH-20, and MCI gel CHP-20P columns) to isolate one novel alkaloid (**1**, pale-brown powder, 68 mg), nine known alkaloids (rhynchophylloside G (**2**) [5], vincosamide 11-*O*-*β*-D-glucopyranoside (**3**) [7], vincosamide (**4**) [8], strictosamide (**5**) [9], 5*b*-carboxystrictosidine (**6**) [10], strictosidine (**7**) [11], cadambine (**8**) [11], 3*a*-dihydrocadambine (**9**) [12], and (*E*)-vallesiachotamine (**10**) [13]), and thirteen known non-alkaloids (8-epiloganic acid (**11**) [14], chlorogenic acid (**12**) [15], erigeside C (**13**) [16], (1*S*,2*R*,3*S*)-lyoniresinol-3*a*-O-*β*-glucoside (**14**) [17], (1*R*,2*S*,3*R*)-lyoniresinol-3*a*-O-*β*-glucoside (**15**) [17], ( +)-catechin (**16**) [18], (−) -epicatechin (**17**) [18], hyperin (**18**) [19], rutin (**19**) [20], procyanidin B1 (**20**) [21], procyanidin B2 (**21**) [21], procyanidin B3 (**22**) [21], and procyanidin B4 (**23**) [21]) (Figs. 1 and 2).

Compound **1** was obtained as a pale-brown powder and its molecular formula was estimated as C_38_H_48_N_2_O_20_ by high resolution electrospray ionization mass spectrometry (HR-ESI–MS) at *m/z* 875.2698 [M + Na]^+^ (calculated for C_38_H_48_N_2_O_20_Na, 875.2693). The ^1^H, ^13^C and DEPT NMR spectra (Table 1, Figs. S4, S5) as well as HSQC spectra (Fig. S6) implied the presence of nineteen sp^3^ methines, four sp^2^ methines, five sp^3^ methylenes, six sp^2^ quaternary carbons, one vinyl group [*δ*_C_ 133.2 (C-19); *δ*_H_ 5.47 (1H, dt, *J* = 17.1, 8.6 Hz, H-19), *δ*_C_ 120.6 (C-18); *δ*_H_ 5.21 (1H, dd, *J* = 8.6, 1.9 Hz, H-18), and *δ*_H_ 5.33 (1H, dd, *J* = 17.1, 1.9 Hz, H-18)], two carbonyl groups [*δ*_C_ 162.1(C-22) and 172.9 (C-7)] and three anomeric protons [*δ*_H_ 4.43 (1H, d, *J* = 7.6 Hz, H-1′′), 4.69 (1H, d, *J* = 7.6 Hz, H-1′), 5.00 (1H, d, *J* = 7.3 Hz, H-1′′′)].

**Table 1:** ^1^H and ^13^C NMR data of compounds **1** and rhynchophylloside L (*δ* in ppm)

	1		Rhynchophylloside L	
No	*δ*_C_	*δ*_H_ (*J *in Hz)	*δ*_C_	*δ*_H_ (*J *in Hz)
2	150.3		149.3	
3	60.8	5.10-5.15, m	60.4	5.07-5.10, m
5	48.5	4.22, d (14.0)	48.1	4.19, d (14.3)
		4.72, br.d (14.0)		4.69, br.d (14.0)
6	113.2		112.2	
7	172.9		172.6	
8	121.0		118.8	
9	127.0	8.05, d (8.9)	126.7	7.95, d (8.8)
10	114.5	7.04, dd (8.9, 2.2)	113.9	6.79, br.d (8.8)
11	160.2		160.4	
12	103.4	7.13, d (2.2)	101.5	6.88, br.s
13	142.3		142.3	
14	29.5	1.36, q, (12.3)	29.0	1.33, q (12.2)
		2.45-2.50, m		2.45-2.51, m
15	27.9	3.15-3.20, m	27.5	3.16-3.19, m
16	107.6		107.2	
17	146.9	7.32, d (2.2)	146.5	7.31, s
18	120.6	5.21, dd (8.6, 1.9)	120.1	5.19, br.d (10.5)
		5.33, dd (17.1, 1.9)		5.30, br.d (17.0)
19	133.2	5.47, dt (17.1, 8.6)	132.8	5.46, dt (17.0, 10.5)
20	43.1	2.74, dd (9.5, 6.2)	42.6	2.73, m
21	95.4	5.43, d (1.5)	94.9	5.42, br.s
22	162.1		161.7	
21-O-glc				
1'	96.4	4.69, d (7.6)	96.0	4.69, d (7.4)
2'	80.2	3.38-3.49, m	79.8	3.38-3.41, m
3'	76.8	3.38-3.49, m	76.4	3.41-3.45, m
4'	70.3	3.07-3.14, m	69.9	3.08-3.13, m
5'	77.6	3.20-3.26, m	77.2	3.22, m
6'	61.4	3.38-3.49, m	60.9	3.40-3.47, m
		3.67-3.74, m		3.71, dd (10.6, 5.3)
2'-O-glc.				
1"	103.7	4.43, d (7.6)	103.3	4.43, d (7.6)
2"	75.1	2.96, t (7.3)	74.7	2.96, m
3"	76.8	3.07-3.14, m	76.4	3.08-3.13, m
4"	70.3	2.99-3.06, m	69.9	3.01-3.04, m
5"	77.5	2.99-3.06, m	77.0	3.01-3.04, m
6"	61.6	3.38-3.49, m	61.2	3.40-3.47, m
		3.65, dd (11.1, 5.0)		3.64, dd (10.6, 3.0)
11-O-glc.				
1'''	100.8	5.00, d (7.3)		
2'''	73.6	3.26-3.37, m		
3'''	76.9	3.26-3.37, m		
4'''	69.8	3.20-3.26, m		
5'''	77.6	3.26-3.37, m		
6'''	60.9	3.54, dd (11.8, 5.8)		
		3.67-3.74, m		

**Fig. 3:**
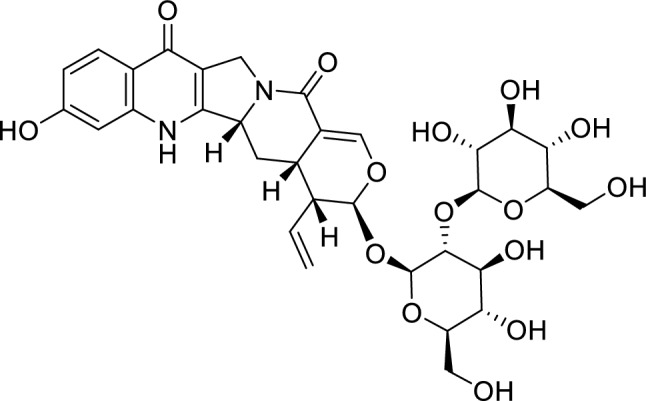
The structure of rhynchophylloside L

**Fig. 4:**
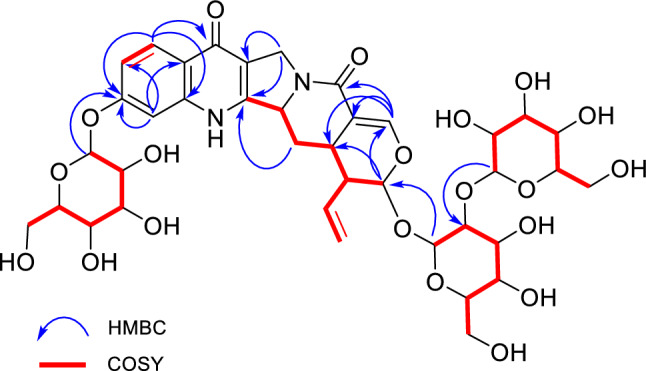
Key HMBC and ^1^H-.^1^H COSY correlations of compound **1**

Chemical shift values in the ^1^H and ^13^C NMR spectra of **1** well resembled those of rhynchophylloside L, except for positions 10 and 12 (Table 1 and Fig. 3), while the estimated molecular formula of **1** (C_38_H_48_N_2_O_20_) corresponded to an addition of C_6_H_10_O_5_ to rhynchophylloside L (C_32_H_38_N_2_O_15_). The COSY and HSQC-TOCSY correlations also supported the presence of an additional spin system in **1** corresponding to an aldohexose moiety (Figs. 4, S8 and S9). The HMBC spectrum displayed long-range ^1^H-^13^C correlations from H-1′ to C-21, H-1′′ to C-2′, and from H-1′′′ to C-11, suggesting that compound **1** shared the same disaccharide chain with rhynchophylloside L at position 21 while the additional sugar is attached to the hydroxy group at C-11 (Figs. 4 and S7). However, the sugar species and their locations in **1** could not be confirmed by the extensive NMR analysis. The nine known alkaloids **2**–**10** and the thirteen known non alkaloids **11**–**23** were identified by comparing their ^1^H and ^13^C NMR spectra and MS data with those reported previously.

**Fig. 5:**
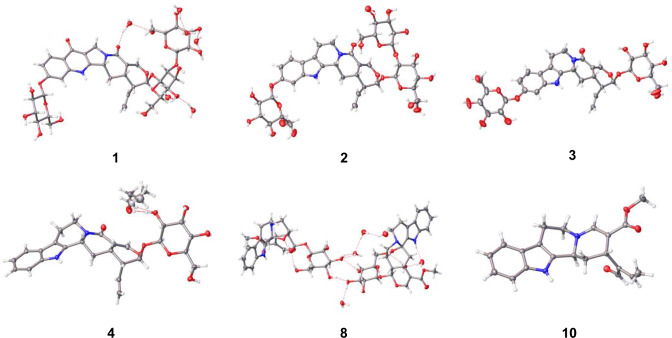
Single-crystal X-ray diffraction analysis of **1**, **2**, **3**, **4**, **8** and **10**

The absolute configurations of **1**, **2**, **3**, **4**, **8**, and **10** were determined by SC-XRD (Cu *K*_α_ radiation) with a Flack parameter (Fig. 5). Given that the initial recrystallization of **1** (1 mg) from water (0.1 mL) afforded very small single crystals (Fig. S14), we reduced the volume of water and obtained better crystals using a micro-drop technique. Specifically, after compound **1** (50 µg) was charged at the center of a V-shaped vial, water (4 µL) was added, and the vial was capped. Use of V-shaped vials is preferred to keep the droplets spherical. The vial was heated to obtain a homogenous solution, with suitable single crystals of **1** subsequently obtained within a day (Fig. S15). This method was applied to grow single crystals of **2** and **3** as well as different solvent systems because absolute configurations of these compounds had been deduced from NMR and CD spectroscopic analyses in the previous reports [5, 7] while their three-dimensional structures remained elusive. The micro-drop crystallization technique allowed us to obtain the first crystallographic data of **2** and **3** only using 50 µg of materials and confirm their proposed stereochemistry were correct.

The micro-drop crystallization method developed in this study forms single crystals without oil; thus, crystallization studies are repeatable with various solvent systems in a single vial and compounds are easily recoverable after the structural analysis. Whereas state-of-the-art techniques such as the nanodrop crystallization method provides higher quality of crystals from smaller amount of molecules [22], our method is sufficient to elucidate the three-dimensional structures of rare natural products at a laboratory scale only using < 100 µg materials and a conventional XRD device, and thus does not require any expensive handling robot or specific environment.

The potential pharmacological activities of *U. rhynchophylla* extracts to treat Alzheimer’s disease are relevant to its inhibition of acetylcholine esterase (AChE) activities in the brain [23]. Monoterpenoid indole alkaloids such as geissoschizine methyl ether N-oxide and geissoschizine methyl ether in this plant exhibit AChE inhibitory activities [24, 25]. We examined in vitro AChE inhibitory activities of all isolated compounds, but no compound showed any inhibitory activity at 200 µM. This result is comparable with the previous report that the glycosylated derivatives of such indole alkaloids rarely show AChE inhibitory activities [5].

## Conclusion

One new indole alkaloid triglucoside (**1**), nine known alkaloids (**2**–**10**), and thirteen known nonalkaloids (**11**–**23**) were isolated from the aqueous extract of *U. rhynchophylla* hooks. The typically difficult-to-obtain single crystals of alkaloid glucosides were prepared using a micro-drop crystallization method. Given the importance of knowing absolute configurations for medicinal applications, we further plan to investigate whether this method can be applied to other compounds reluctantly forming single crystals.

## Experimental

### General experimental procedures

1D and 2D NMR spectra were recorded on a Bruker AVANCE NEO 600 (^1^H: 600 MHz, ^13^C: 150 MHz) spectrometer and JEOL ECA-600 (^1^H: 600 MHz, ^13^C: 150 MHz), using TMS as an internal standard. Chemical Shifts (*δ*) are presented in ppm and coupling constants (*J*) in Hz. HR-ESI–MS experiments were acquired using an Orbitrap Exploris 120 mass spectrometer (Thermo Fisher Scientific). Single-crystal X-ray diffraction data were acquired on a Rigaku XtaLAB Synergy-R diffractometer using Cu Ka radiation. Optical rotation value was recorded on a JASCO P-2200 polarimeter. UV spectra was obtained with a JASCO V-750 spectrophotometer. IR spectra was obtained with a JASCO FT/IR-4600 spectrophotometer. Circular dichroism was measured on JASCO J-1100 spectrometer. Powdered DIAION (Mitsubishi Chemical Co., Japan), Sephadex LH-20 (Amersham Pharmacia Biotech AB, Uppsala, Sweden), MCI gel CHP20P (Mitsubishi Chemical Co., Japan), silica gel 60 (Merck, Darmstadt, Germany) and ODS-A-HG (YMC, Kyoto, Japan, 50 µm) were used for column chromatography (CC). Silica gel GF_254_ plates (Merck; 0.25 mm in thickness) were used for TLC analysis.

### Plant material

The hook-bearing stems of *Uncaria rhynchophylla* (Miq.) Miq. were purchased from Santai Country Yuanhui Commerce and Trade Co., Ltd. in Sichuan, China. A voucher specimen (THS102306) was deposited in the herbarium of Tsumura Co., Ltd. in Japan.

### Isolation of new compound 1

The dried hook-bearing stems of *U. rhynchophylla* (14.25 kg) were percolated with 60% MeOH in H_2_O (101.5 L). After concentration of MeOH, the crude extract in water was partitioned with CHCl_3_ to obtain the CHCl_3_-soluble extract and the water-soluble extract. After concentration of the aqueous phase, the crude extract was subjected to DIAION HP-20 eluting successively with 60% MeOH in H_2_O and MeOH to obtain two fractions (60 M, DM2).

60 M was fractionated by Sephadex LH-20 CC (10–100% MeOH in H_2_O) to afford fractions 60M1 (61.82 g), 60M2 (21.26 g), 60M3 (115.54 g). Further separation of 60M1 (61.82 g) by ODS CC (10–100% MeOH in H_2_O) to obtain five fractions 60M11, 60M12, 60M13, 60M14 and 60M15. 60M13 was chromatographed on silica gel CC (CHCl_3_/MeOH/H_2_O, 40:10:1–6:4:1, v/v, successively) to obtain fractions 60M131, 60M132 and 60M133. Purification of 60M133 on ODS CC (15–16% MeCN in 50 mM NH_4_Ac aq.) and MCI gel CHP-20P (H_2_O to MeOH) afford compound **1** (68 mg). The isolation scheme of other known compounds is summarized in supplementary Fig. S13.

### Spectroscopic data of rhynchophylloside L 11-O-β-D-glucopyranoside (1)

Pale-brown powder; [*α*]_D_^20^ –99 (*c* = 0.1, MeOH); UV λ_max_ (MeOH) nm (log ɛ): 247 (4.71), 253 (4.71), 310 (4.07), 321 (4.06); CD λ_max_ (MeOH) nm (Δɛ): 224 (+ 10.47), 252 (–11.09), 294 (+ 1.02); IR (KBr) cm^–1^: 3398, 2923, 2877, 1635, 1604, 1574, 1454, 1250, 1076, 505; ^1^H and ^13^C NMR (600 and 150 MHz, respectively, DMSO-*d*_6_), see Table 1; positive ion HR-ESI–MS, *m/z* 875.2698 [M + Na]^+^ (calculated for C_38_H_48_N_2_O_20_Na, 875.2693).

### SC-XRD analysis of 1, 2, 3, 4, 8 and 10

Compound **1** (50 µg) was placed into V-type vial and added 4 µL of water. After caped vial, the suspension was dissolved by heating. The suitable single crystals of compound **1** was crystalized within a day. (C_38_H_48_N_2_O_20_)·6(H_2_O), *M* = 960.88, crystal size 0.142 × 0.013 × 0.008 mm^3^, orthorhombic, space group *P*2_1_2_1_2_1_, *a* = 6.9177(1) Å, *b* = 21.6692(3) Å, *c* = 28.7263(5) Å, V = 4306.10(11) Å ^3^, Z = 4, *α* = *β* = *γ* = 90°, density (calcd) = 1.482 g·cm^–3^, F(000) = 2040.0, reflections collected/unique 64,432/8602 (*R*_int_ = 0.0467), final R indices (*I* > 2σ (*I*)) *R*_1_ = 0.0476, *wR*_2_ = 0.1043, goodness of fit = 1.149, Flack parameter = 0.01(5). Crystallographic data for compound **1** have been deposited with the Cambridge Crystallographic Data Center (CCDC 2258683).

Compound **2** (50 µg) was placed into V-type vial and added 10 µL of water/ethanol/acetonitrile. After caped vial, the suspension was dissolved by heating. The suitable single crystals of compound **2** was crystalized within a day. (C_38_H_50_N_2_O_19_)·2(H_2_O), *M* = 874.83, crystal size 0.19 × 0.04 × 0.03 mm^3^, orthorhombic, space group *P*2_1_2_1_2_1_, *a* = 5.8287(2) Å, *b* = 19.4840(4) Å, *c* = 38.4044(10) Å, V = 4361.4(2) Å ^3^, Z = 4, *α* = *β* = *γ* = 90°, density (calcd) = 1.332 g·cm^–3^, F(000) = 1856.0, reflections collected/unique 37,841/8418 (*R*_int_ = 0.0458), final R indices (*I* > 2σ (*I*)) *R*_1_ = 0.0557, *wR*_2_ = 0.1514, goodness of fit = 1.125, Flack parameter = 0.09(7). Crystallographic data for **2** have been deposited with the Cambridge Crystallographic Data Center (CCDC 2264009).

Compound **3** (50 µg) was placed into V-type vial and added 10 µL of water/acetonitrile. After caped vial, the suspension was dissolved by heating. The suitable single crystals of compound **3** was crystalized within a day. C_32_H_40_N_2_O_14_, *M* = 676.66, crystal size 0.05 × 0.03 × 0.01 mm^3^, monoclinic, space group *P*2_1_, *a* = 16.8414(6) Å, *b* = 5.9860(2) Å, *c* = 18.8582(6) Å, V = 1881.77(11) Å ^3^, Z = 2, *α* = *γ* = 90°, *β* = 98.187(3)°, density (calcd) = 1.194 g·cm^–3^, F(000) = 716.0, reflections collected/unique 23,514/7326 (*R*_int_ = 0.0507), final R indices (*I* > 2σ (*I*)) *R*_1_ = 0.0607, *wR*_2_ = 0.1521, goodness of fit = 1.019, Flack parameter = − 0.2(2). Crystallographic data for **3** have been deposited with the Cambridge Crystallographic Data Center (CCDC 2263997).

Compound **4** (50 µg) was placed into V-type vial and added 10 µL of ethanol. After caped vial, the suspension was dissolved by heating. The suitable single crystals of compound **4** was crystalized within a day. (C_26_H_30_N_2_O_8_)·(C_2_H_6_O), *M* = 544.59, crystal size 0.25 × 0.02 × 0.014 mm^3^, monoclinic, space group *P*2_1_, *a* = 8.2300(2) Å, *b* = 5.85790(10) Å, *c* = 27.3883(6) Å, V = 1307.99(5) Å ^3^, Z = 2, *α* = *γ* = 90°, *β* = 97.862(2), density (calcd) = 1.383 g·cm^–3^, F(000) = 580.0, reflections collected/unique 26,131/5286 (*R*_int_ = 0.0484), final R indices (*I* > 2σ (*I*)) *R*_1_ = 0.0469, *wR*_2_ = 0.1305, goodness of fit = 1.097, Flack parameter = 0.00(10). Crystallographic data for **4** have been deposited with the Cambridge Crystallographic Data Center (CCDC 2263990).

Compound **8** was recrystallized from methanol. 2(C_27_H_32_N_2_O_10_)·8(H_2_O), *M* = 1233.22, crystal size 0.12 × 0.05 × 0.03 mm^3^, orthorhombic, space group *P*2_1_2_1_2_1_, *a* = 12.7952(4) Å, *b* = 19.1742(9) Å, *c* = 24.2979(9) Å, V = 5961.2(4) Å ^3^, Z = 4, *α* = *β* = *γ* = 90°, density (calcd) = 1.374 g·cm^–3^, F(000) = 2624.0, reflections collected/unique 39,208/11453 (*R*_int_ = 0.0697), final R indices (*I* > 2σ (*I*)) *R*_1_ = 0.0790, *wR*_2_ = 0.1902, goodness of fit = 1.176, Flack parameter = 0.04(11). Crystallographic data for **8** have been deposited with the Cambridge Crystallographic Data Center (CCDC 2259276).

Compound **10** was recrystallized from acetone. C_21_H_22_N_2_O_3_, *M* = 350.40, crystal size 0.11 × 0.06 × 0.04 mm^3^, orthorhombic, space group *P*2_1_2_1_2_1_, *a* = 7.13640(10) Å, *b* = 9.8793(2) Å, *c* = 25.4299(5) Å, V = 1792.88(6) Å ^3^, Z = 4, *α* = *β* = *γ* = 90°, density (calcd) = 1.298 g·cm^–3^, F(000) = 744.0, reflections collected/unique 17,419/3672 (*R*_int_ = 0.0306), final R indices (*I* > 2σ (*I*)) *R*_1_ = 0.0339, *wR*_2_ = 0.0954, goodness of fit = 0.810, Flack parameter = 0.07(9). Crystallographic data for **10** have been deposited with the Cambridge Crystallographic Data Center (CCDC 2255637).

### AChE Inhibitory Assay

The AChE inhibitory activities of isolated compounds were examined based on Ellman’s method [26] using Amplite^®^ Colorimetric Acetylcholinesterase Assay Kit (AAT Bioquest, Inc.). Briefly, 10 µL of the tested compound solution dissolved in H_2_O with or without 4% DMSO was mixed with 30 µL of the Assay Buffer and 10 µL of acetylcholinesterase solution (200 mU/mL in the Assay Buffer) on a 96-well microplate. Neostigmine bromide was used as a positive control. After incubation at room temperature for 20 min to facilitate binding of compounds to AChE, 50 µL of the Working Solution containing 5,5′-dithiobis(2-nitrobenzoic acid) and acetylthiocholine iodide was added. After 30 min incubation, the absorbance of each well was recorded at a wavelength of 405 nm. Following the previous report [5], the AChE inhibitory activity was calculated as follows: inhibition % = (*C* − *S*)/ (*C* − *B*) × 100% (*C*, the absorbance of control; *S*, the absorbance of sample solution; and *B*, the absorbance of blank).

## Supplementary Information

Below is the link to the electronic supplementary material.

Supplementary file1 (PDF 954 KB)
