# Supplementary material for: Isolation of new indole alkaloid triglucoside from the aqueous extract of Uncaria rhynchophylla
Source: J Nat Med. 2024 Aug 22;79(1):28–35. doi: 10.1007/s11418-024-01836-9 (PMC11735493; doi:10.1007/s11418-024-01836-9)
Supplement: Supplementary file 1 — Supplementary file1 (PDF 954 KB) [file 11418_2024_1836_MOESM1_ESM.pdf]

Supplementary Data

Isolation of New Indole Alkaloid Triglucoside from the Aqueous Extract of *Uncaria Rhynchophylla*

Yuta Koseki, Hiroaki Nishimura, Ryuji Asano, Katsuyuki Aoki, Li shiyu, Ryosuke Sugiyama, Mami Yamazaki

Contents:

Figure S1: HR-ESI-MS of compound **1**
Figure S2: ^{1}H NMR of compound **1** in DMSO-d_6 (600 MHz)
Figure S3: ^{13}C NMR of compound **1** in DMSO-d_6 (150 MHz)
Figure S4: ^{13}C DEPT135 NMR of compound **1** in DMSO-d_6 (150 MHz)
Figure S5: ^{13}C DEPT90 NMR of compound **1** in DMSO-d_6 (150 MHz)
Figure S6: HSQC of compound **1** in DMSO-d_6 (600 and 150 MHz)
Figure S7: HMBC of compound **1** in DMSO-d_6 (600 and 150 MHz)
Figure S8: DQF-COSY of compound **1** in DMSO-d_6 (600 and 150 MHz)
Figure S9: HSQC-TOCSY of compound **1** in DMSO-d_6 (600 and 150 MHz)
Figure S10: UV of compound **1**
Figure S11: IR of compound **1**
Figure S12: ECD of compound **1**
Figure S13: Extraction and isolation of **1-23**
Figure S14: The picture of tiny single crystals of compound **1**
Figure S15: The picture of suitable single crystals of compound **1**

Figure S1: HR-ESI-MS of compound **1**

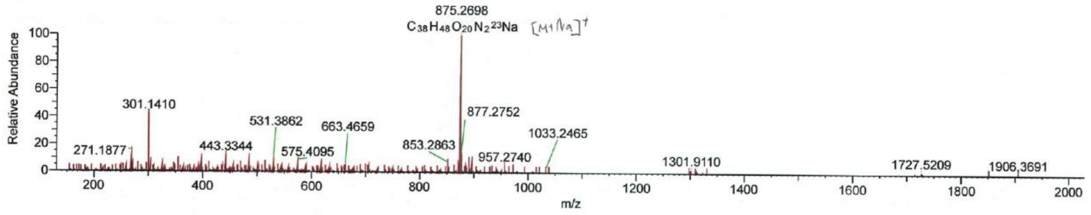

Peak Mass	Display Form...	S Fit	RDB	Delta [ppm]	Theo. mass	Rank	Combined S...	# Matched Iso.	# Missed Iso.	MS Cov. [%]	Pattern Cov....	MSMS Matc...
875.2698	$C_{38}H_{48}O_{20}N_2{}^{23}Na$	47.7647499219518	15.50	0.59	875.26926	1	93.78	5	0	96.34	99.68	(Collection)
875.2698	$C_{40}H_{47}O_{20}N_2$	41.4488109599616	18.50	-2.16	875.27167	2	93.45	5	0	96.34	99.64	(Collection)
875.2698	$C_{50}H_{44}O_{13}{}^{23}Na$	39.6105995236329	28.50	2.70	875.26741	3	92.35	5	0	95.28	97.71	(Collection)

Figure S2: ^{1}H NMR of compound **1** in DMSO-d_6 (600 MHz)

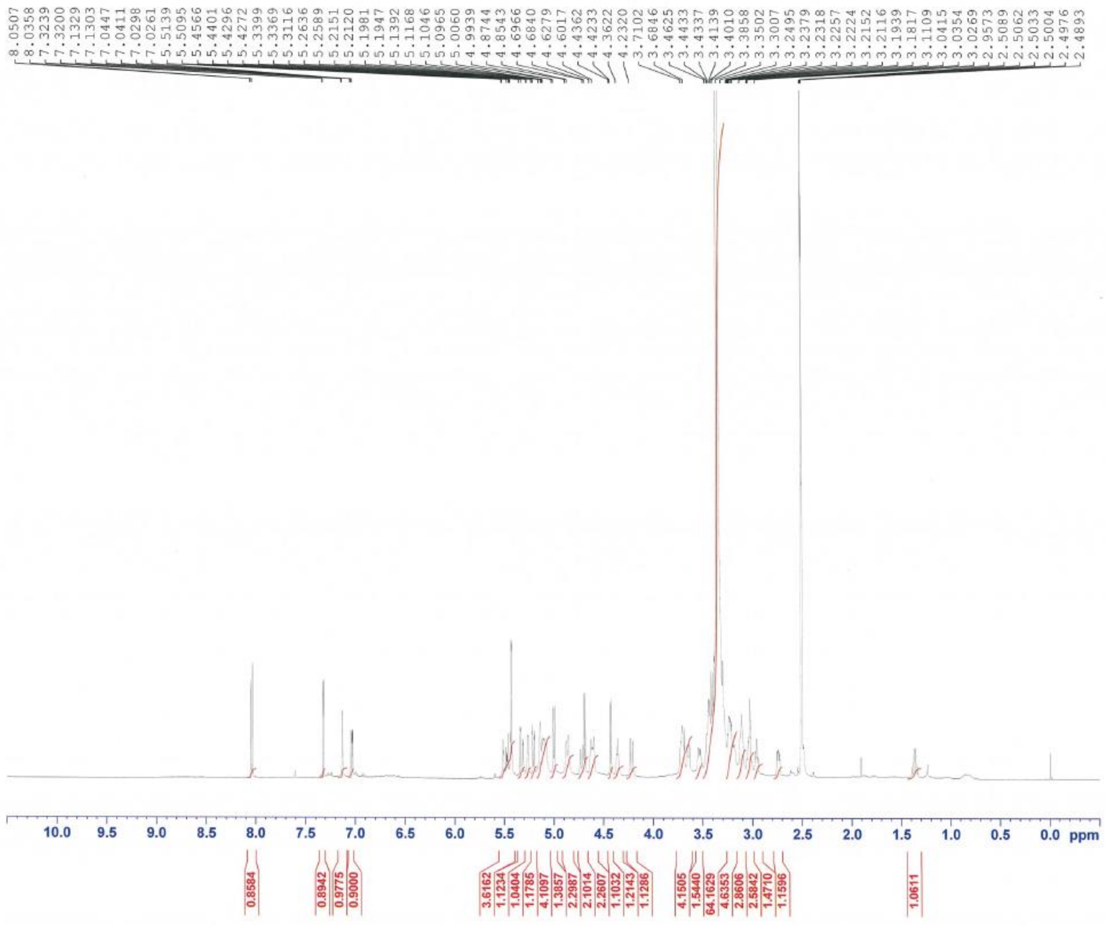

Figure S3: ^{13}C NMR of compound **1** in DMSO-d_6 (150 MHz)

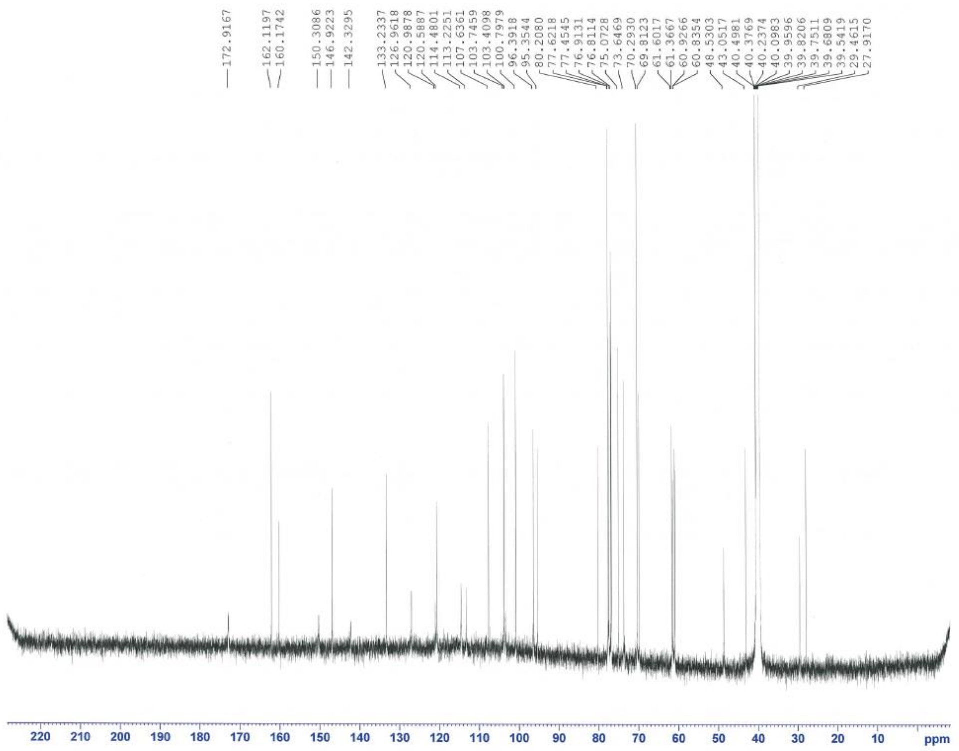

Figure S4: ^{13}C DEPT135 NMR of compound **1** in DMSO-d_6 (150 MHz)

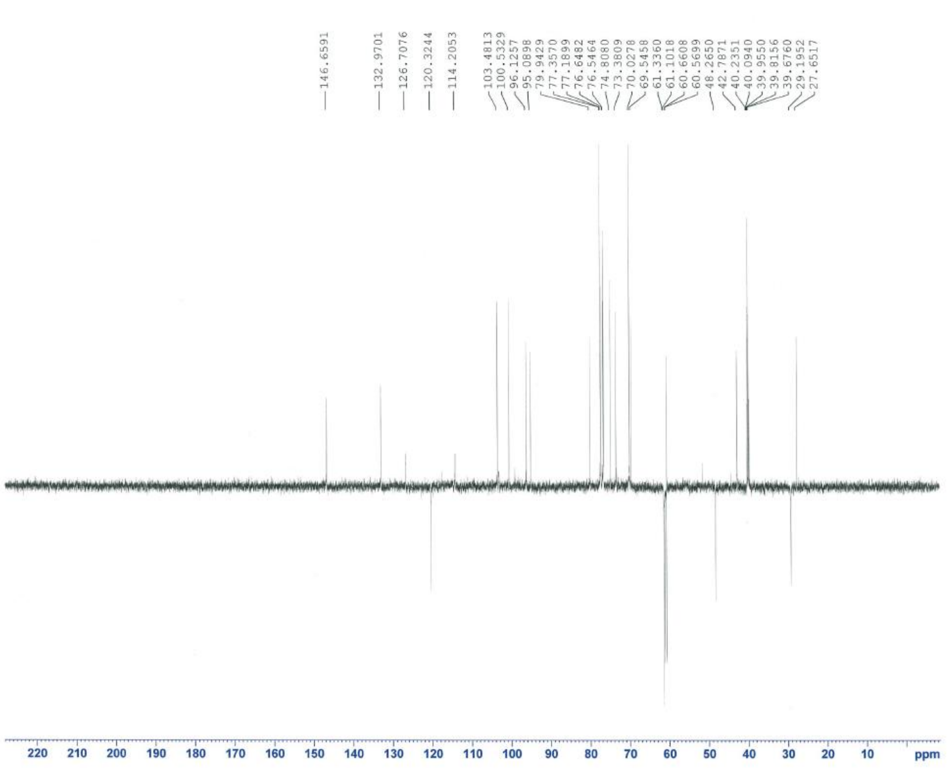

Figure S5: ^{13}C DEPT90 NMR of compound **1** in DMSO-d_6 (150 MHz)

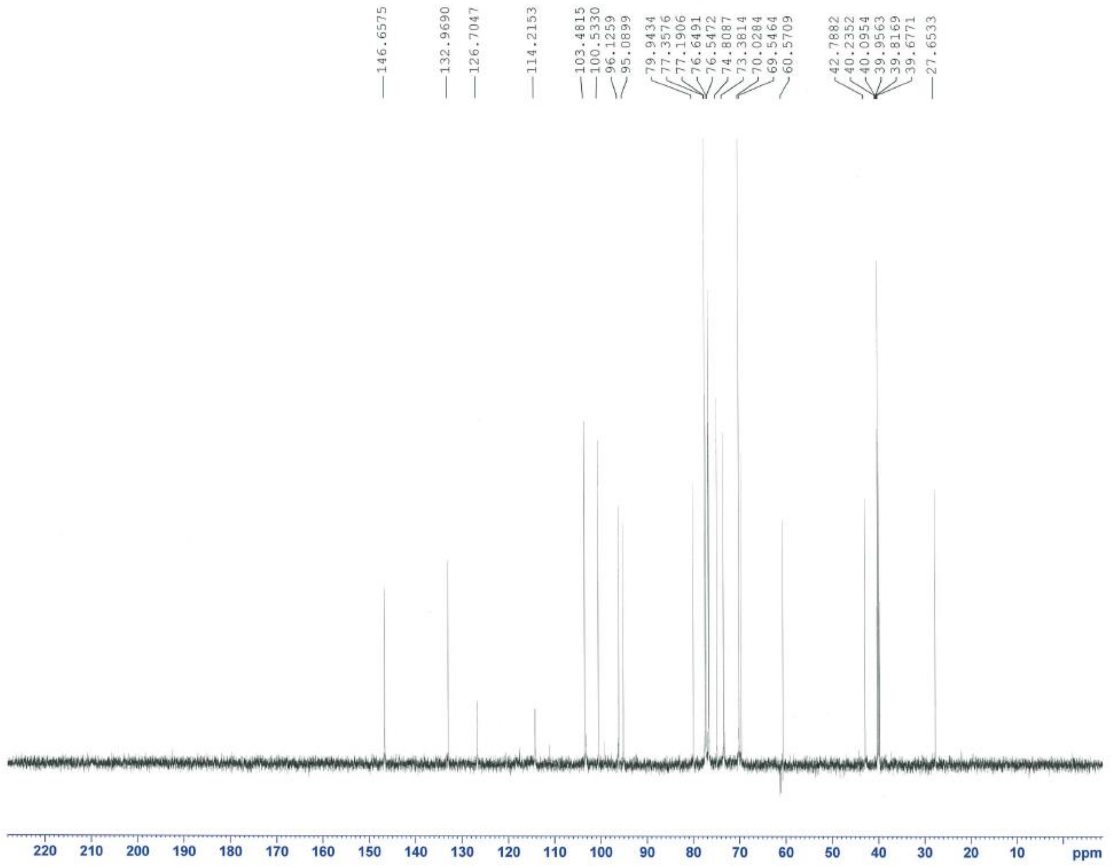

Figure S6: HSQC of compound **1** in DMSO-d_6 (600 and 150 MHz)

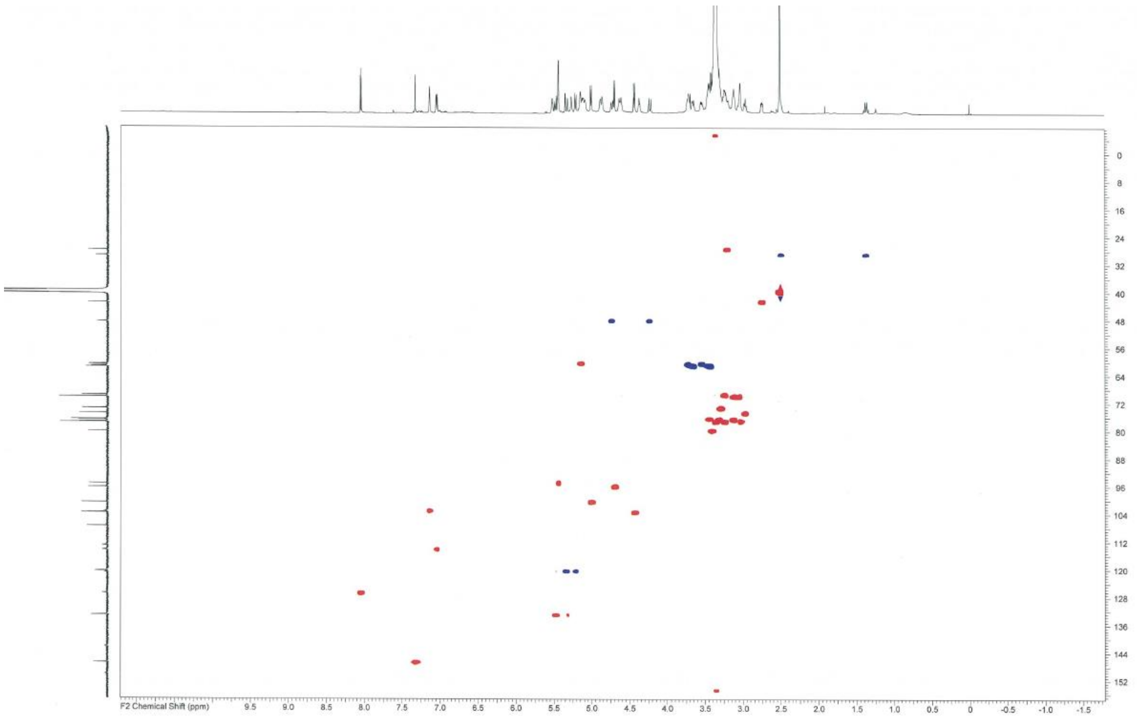

Figure S7: HMBC of compound **1** in DMSO-d_6 (600 and 150 MHz)

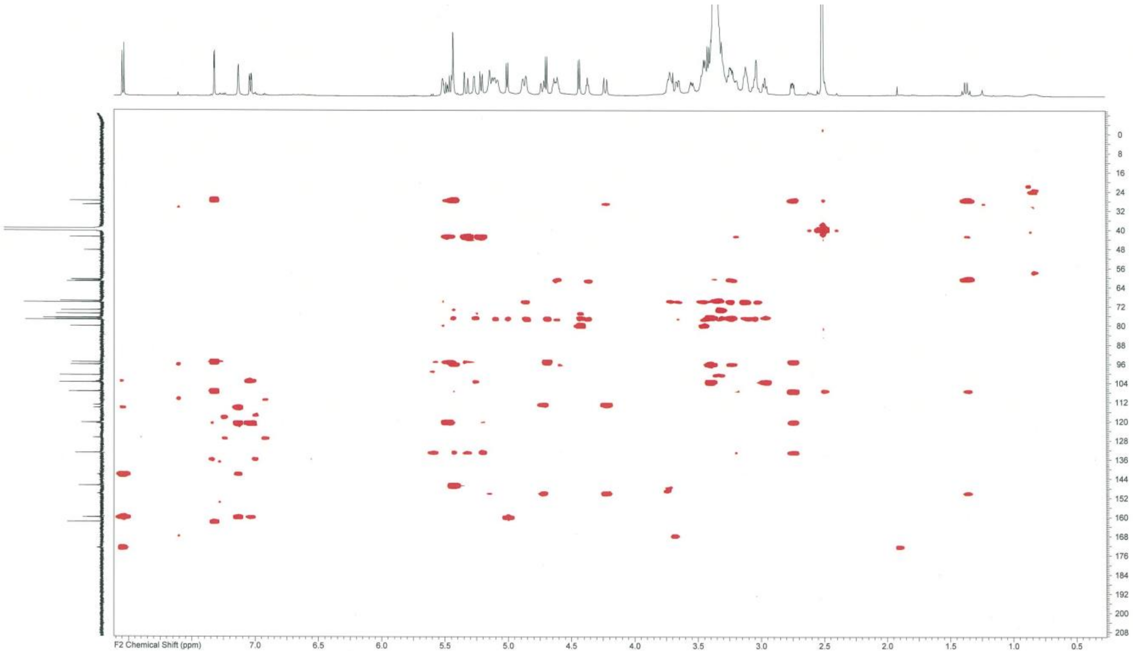

Figure S8: DQF-COSY of compound **1** in DMSO-d_6 (600 and 150 MHz)

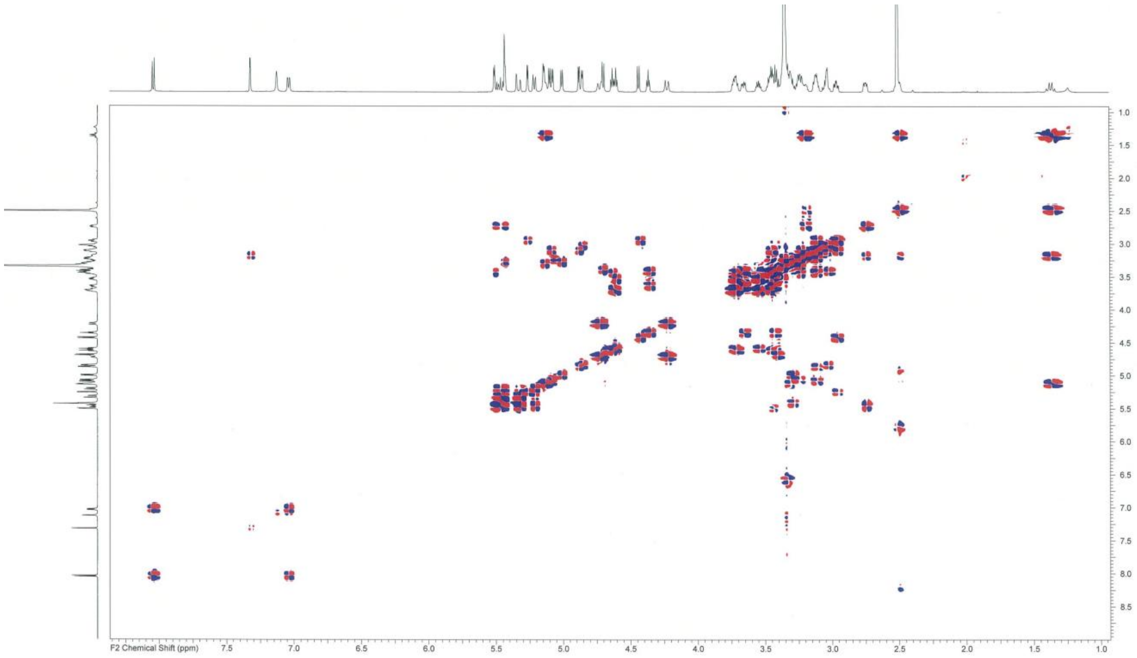

Figure S9: HSQC-TOCSY of compound **1** in DMSO-d_6 (600 and 150 MHz)

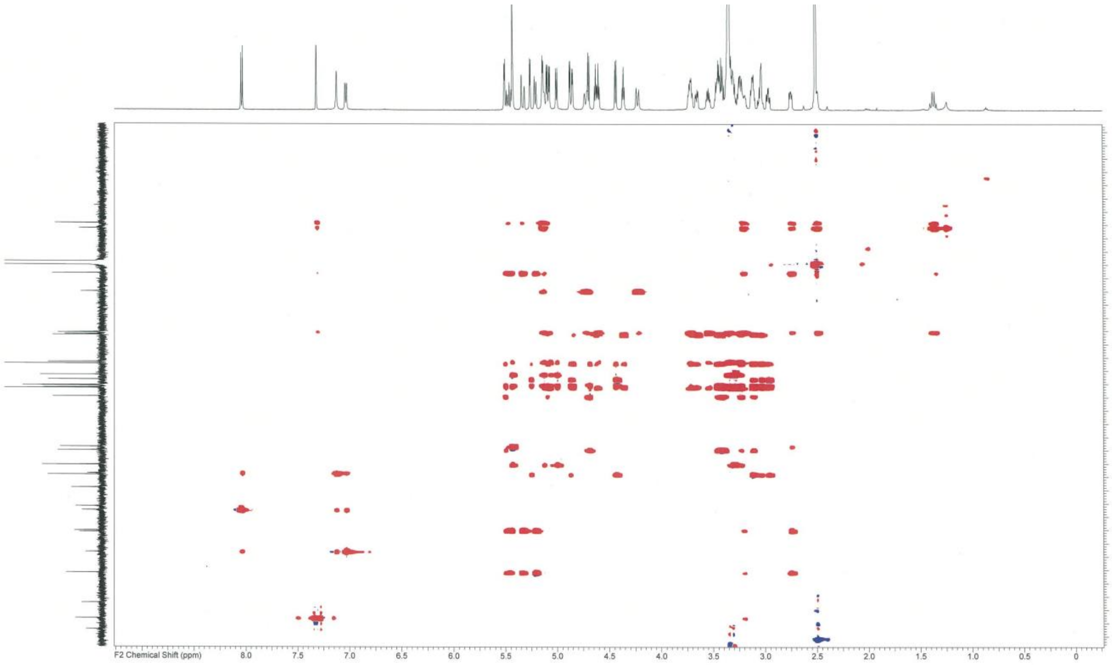

Figure S10: UV of compound **1**

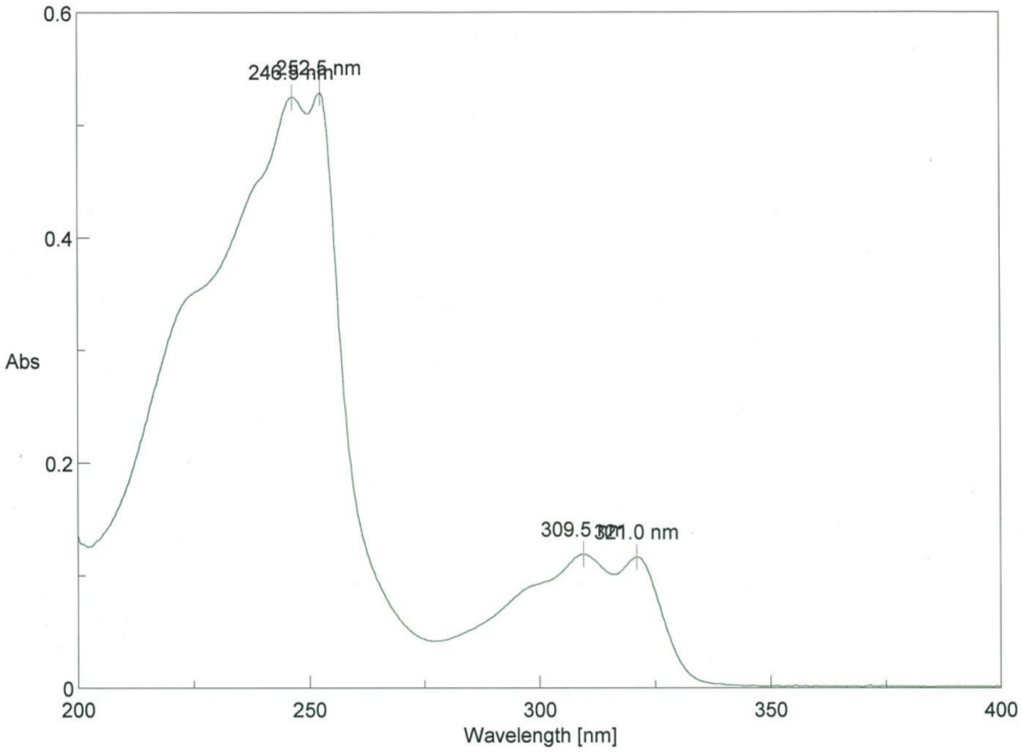

Figure S11: IR of compound **1**

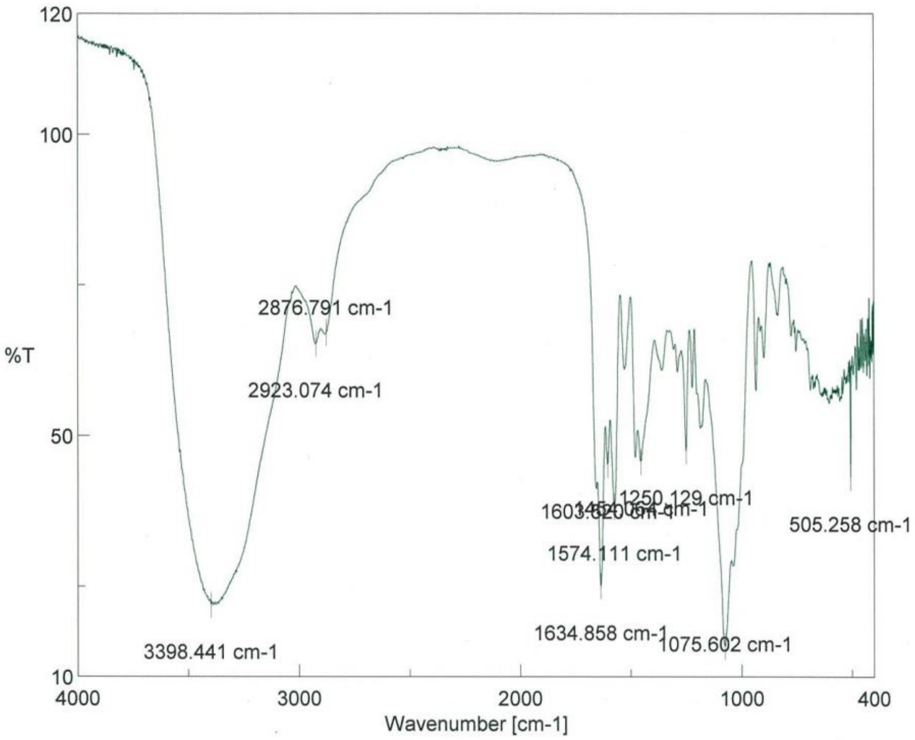

Figure S12: ECD of compound **1**

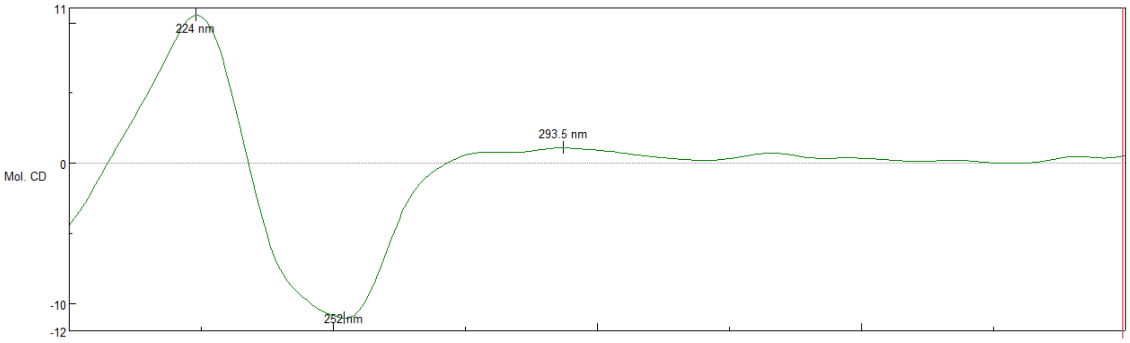

Figure S13: Extraction and isolation of **1-23**

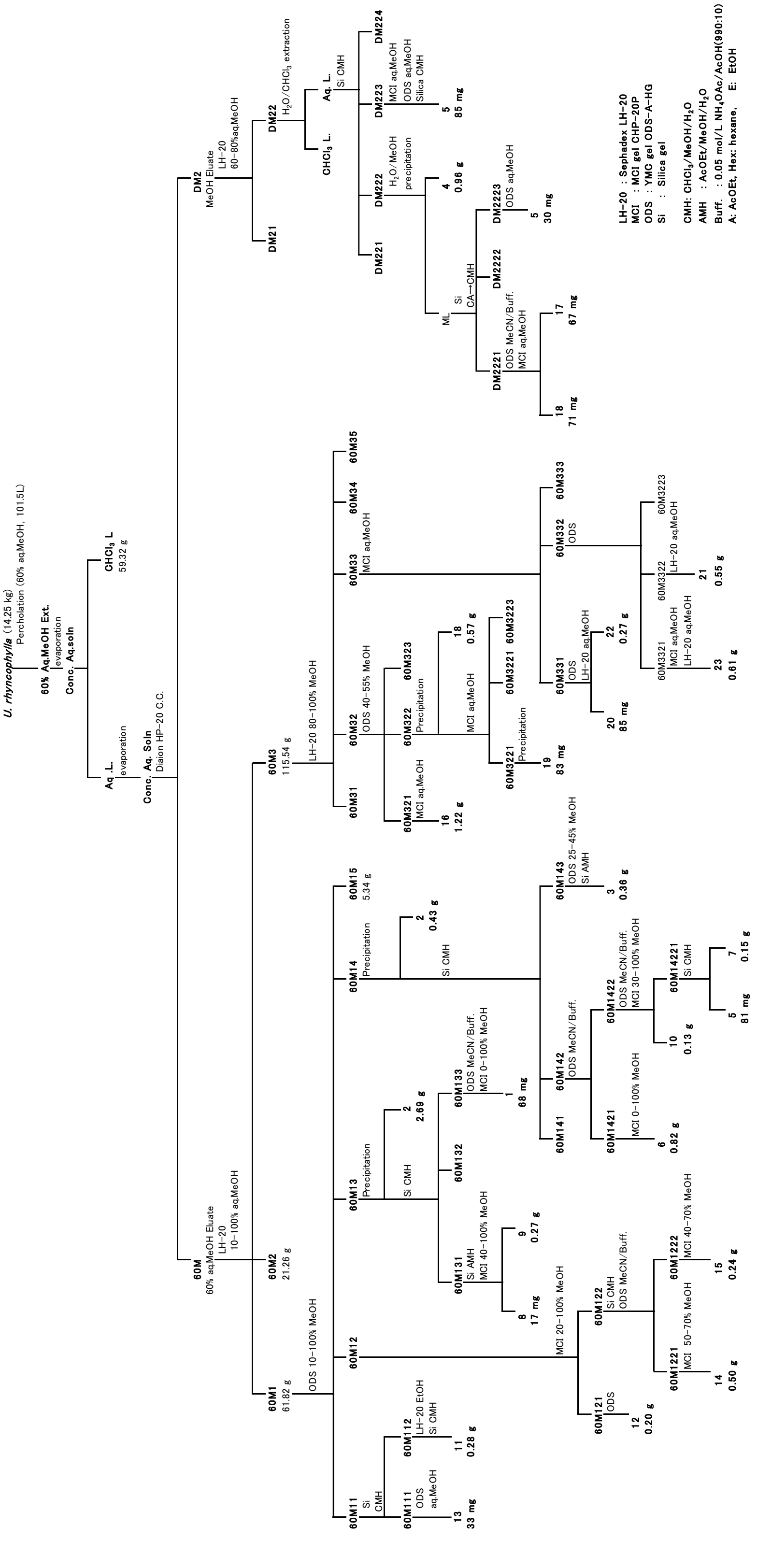

The dried hook-bearing stems of *U. rhynchophylla* (14.25 kg) were percolated with 60% MeOH in H_2O (101.5 L). After concentration of MeOH, the crude extract in water was partitioned with $CHCl_3$ to obtain the $CHCl_3$-soluble extract and the water-soluble extract. After concentration of the water-soluble extract, the crude was subjected to DIAION HP-20 eluting successively with 60% MeOH in H_2O, MeOH to obtain fractions 60M, DM2.

60M was chromatographed by Sephadex LH-20 CC (10-100% MeOH in H_2O) to afford fractions 60M1 (61.82 g), 60M2 (21.26 g), 60M3 (115.54 g). Further separation of 60M1 (61.82 g) by ODS CC (10-100% MeOH in H_2O) to obtain five fractions 60M11, 60M12, 60M13, 60M14 and 60M15. 60M11 was subjected to silica gel CC eluting with a gradient system of AcOEt/MeOH/H_2O (8:2:0-6:4:1, v/v, successively) to afford two fractions 60M111 and 60M112.

Compound **13** (13 mg) was purified from 60M111 by ODS CC (5-25% MeOH in H_2O, v/v, successively). Purification of 60M112 on Sephadex LH-20 (EtOH) and silica gel CC ($CHCl_3$/MeOH/H_2O, 8:2:0.2-6:4:1, v/v, successively) afforded **11** (280 mg). 60M12 was chromatographed on MCI gel CHP-20P (20-100% MeOH in H_2O, v/v, successively) to give fractions 60M121 and 60M122. Compound **12** (200 mg) was obtained from 60M121 by ODS CC (0-5% MeCN in 50 mM NH_4Ac aq.). 60M122 was subjected to silica gel CC ($CHCl_3$/MeOH/H_2O, 68:12:1-14:6:1, v/v, successively) and ODS CC (5-15% MeCN in 50 mM NH_4Ac aq.) to give fractions 60M1221 and 60M1222.

Compound **14** (0.50 g) was purified from 60M1221 by MCI gel CHP-20P (50-70% MeOH in H_2O). Purification of 60M1222 on MCI gel CHP-20P (40-70% MeOH in H_2O) afforded **15** (0.24 g). Compound **2** (2.69 g) was precipitated from fraction 60M13 and the

filtrate was chromatographed on silica gel CC ($CHCl_3$/MeOH/H_2O, 40:10:1-6:4:1, v/v, successively) to obtain fractions 60M131, 60M132 and 60M133. Compound **8** (17 mg) and **9** (0.27 g) was purified by silica gel CC (AcOEt/MeOH/H_2O, 30:2:1-8:2:1, v/v, successively) and MCI gel CHP-20P (40-100% MeOH in H_2O). Purification of 60M133 on ODS CC (15-16% MeCN in 50 mM NH_4Ac aq.) and MCI gel CHP-20P (H_2O to MeOH) afford compound **1** (68 mg). After the filtration of precipitation of compound **2** (0.43 g) in 60M14, the filtrate was subjected to silica gel CC ($CHCl_3$/MeOH/H_2O, 8:2:0.2-6:4:1, v/v, successively) to give fractions 60M141, 60M142, 60M143 and 60M144. 60M142 was chromatographed on ODS (15-20% MeCN in 50 mM NH_4Ac aq.) to obtain fractions 60M1421 and 60M1422. Compound **6** (0.82 g) was purified by MCI gel CHP-20P (H_2O to MeOH) from 60M1421. 60M1422 was subjected to ODS CC (15-25% MeCN in 50 mM NH_4Ac aq.) and MCI gel CHP-20P (30-100% MeOH in H_2O) to give compound **10** (0.13 g) and subfraction 60M14221, from which compound **5** (81 mg) and **7** (0.15 g) was obtained by silica gel CC ($CHCl_3$/MeOH/H_2O, 8:2:0.2-14:6:1, v/v, successively). 60M143 was chromatographed on ODS CC (25-45% MeOH in H_2O) and silica gel CC (AcOEt/MeOH/H_2O, 10:1.5:1-6:4:1, v/v, successively) to give compound **3** (0.36 g).

60M3 (115.54 g) was separated by Sephadex LH-20 (80-100% MeOH in H_2O) to give fractions 60M31, 60M32, 60M33, 60M34 and 60M35. 60M32 was subjected to ODS CC (40-55% MeOH in H_2O) to afford fractions 60M321, 60M322 and 60M323. Compound **16** (1.22 g) was isolated from 60M321 by MCI gel CHP-20P (20-100% MeOH in H_2O). Compound **18** (0.57 g) was collected from the precipitation of 60M322 and the filtrate was chromatographed on MCI gel CHP-20P (20-100% MeOH in H_2O) to give fractions 60M3221, 60M3222 and 60M3223. Compound **19** (83 mg) was purified by Sephadex

LH-20 (60-80% MeOH in H_2O) from 60M3221. 60M33 was subjected to MCI gel CHP-20P (20-50% MeOH in H_2O) to give fractions 60M331, 60M332 and 60M333. Compound **20** (85 mg) and **22** (0.27 g) were isolated from 60M331 by ODS (0.5% AcOH in H_2O /MeOH, 95:5-90:10, v/v, successively) and Sephadex LH-20 (60% MeOH in H_2O). 60M332 was chromatographed on ODS (0.5% AcOH in H_2O /MeOH, 92:8-88:12, v/v, successively) to afford fractions 60M3321, 60M3322 and 60M3323. 60M3321 was purified by MCI gel CHP-20P (20-30% MeOH in H_2O) and Sephadex LH-20 (60% MeOH in H_2O) to give Compound **23** (0.61 g). Compound **21** (0.55 g) was obtained from 60M3322 by Sephadex LH-20 (60% MeOH in H_2O).

DM2 was subjected to Sephadex LH-20 (60-80% MeOH in H_2O) to afford fractions DM21 (19.49 g) and DM22 (9.97 g). DM22 dissolved in H_2O was partitioned with $CHCl_3$ to obtain water-soluble extract. The extract was chromatographed on silica gel CC ($CHCl_3$/MeOH/H_2O, 40:10:1-6:4:1, v/v, successively) to give fractions DM221, DM222, DM223 and DM224. Compound **4** (0.96 g) was collected from the precipitation of DM222, and the filtrate was chromatographed on silica gel CC ($CHCl_3$/MeOH/H_2O, 180:20:1-14:6:1, v/v, successively) to give fractions DM2221, DM2222 and DM2223. DM2221 was purified by ODS CC (5-15% MeCN in 50 mM NH_4Ac aq.) and MCI gel CHP-20P (50-70% MeOH in H_2O) to give compound **17** (67 mg) and compound **18** (71 mg). Compound **5** (30 mg) was isolated from DM2223 by ODS CC (60-80% MeOH in H_2O). DM223 was subjected to MCI gel CHP-20P (50-60% MeOH in H_2O), ODS CC (60-70% MeOH in H_2O) and silica gel CC ($CHCl_3$/MeOH/H_2O, 180:25:1-70:12:1, v/v, successively) to give compound **5** (85 mg).

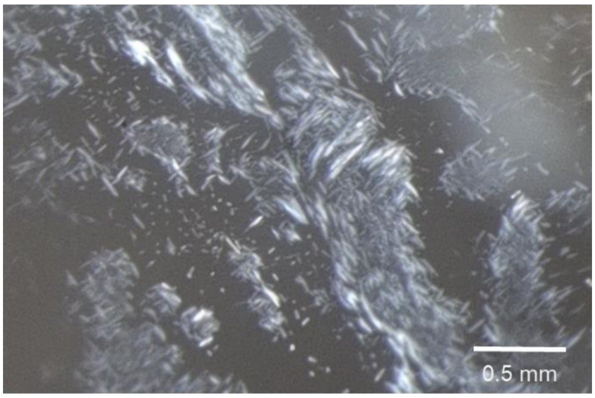

Figure S14: The picture of tiny single crystals of compound **1**

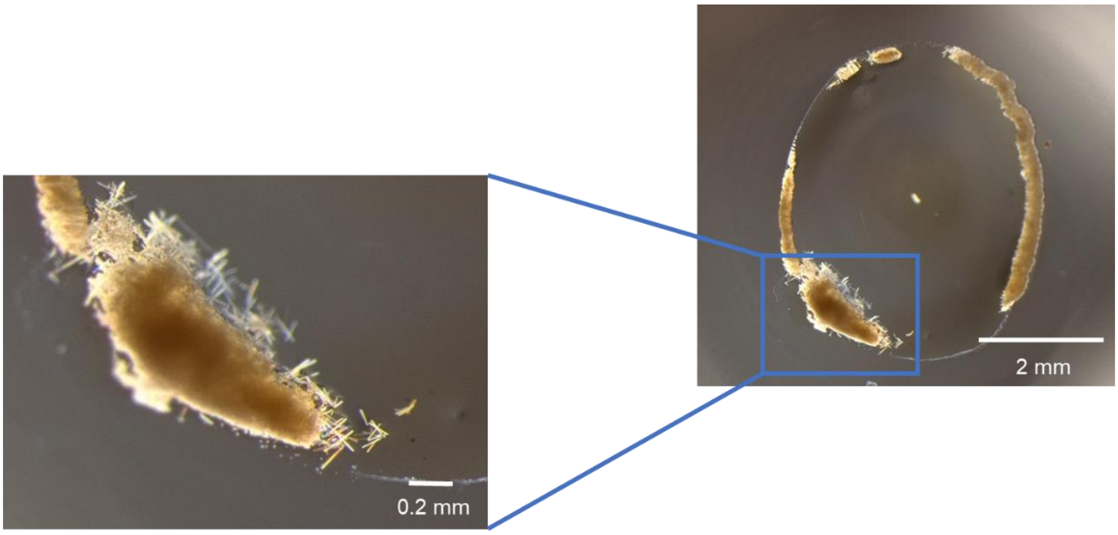

Figure S15: The picture of suitable single crystals of compound **1**
